# *PUF60/AURKA* Axis Contributes to Tumor Progression and Malignant Phenotypes in Bladder Cancer

**DOI:** 10.3389/fonc.2020.568015

**Published:** 2020-10-07

**Authors:** Qian Long, Xin An, Miao Chen, Nan Wang, Silei Sui, Yixin Li, Changlin Zhang, Kaping Lee, Xiaonan Wang, Tian Tian, Yangxun Pan, Huijuan Qiu, Fangyun Xie, Wuguo Deng, Fufu Zheng, Liru He

**Affiliations:** ^1^Sun Yat-sen University Cancer Center, State Key Laboratory of Oncology in South China, Collaborative Innovation Center for Cancer Medicine, Guangzhou, China; ^2^College of Life Science, Jiaying University, Meizhou, China; ^3^Institute of Cancer Stem Cell, Dalian Medical University, Dalian, China; ^4^The First Affiliated Hospital, Zhengzhou University, Zhengzhou, China; ^5^The Seventh Affiliated Hospital, Sun Yat-sen University, Shenzhen, China; ^6^The First Affiliated Hospital, Sun Yat-sen University, Guangzhou, China

**Keywords:** *PUF60*, *AURKA*, bladder cancer, transcriptional regulation, biomarker

## Abstract

Abnormal expression or mutation of RNA splicing proteins are widely observed in human cancers. Here, we identified poly(U) binding splicing factor 60 (*PUF60*) as one of the most differentially expressed genes out of 97 RNA splicing proteins between normal and bladder cancer tissues by bioinformatics analysis of TCGA bladder cancer expression data. The expression of *PUF60* was significantly higher in tumor tissues, while high *PUF60* expression was associated with malignant phenotypes of bladder cancer and shorter survival time. Moreover, we identified aurora kinase A (*AURKA*) as a new downstream target of *PUF60* in bladder cancer cells. *PUF60* knockdown significantly inhibited cell viability and colony formation capacity in bladder cancer cells, whereas *AURKA* overexpression reversed this inhibition effect. Overexpression of *PUF60* significantly promoted cell viability and colony formation in bladder cancer cells, while treatment with *AURKA* specific inhibitor reversed this promotive effect. Mechanistically, *PUF60* specifically bound to the *AURKA* promoter, thereby activating its transcription and expression. Furthermore, we showed that there was a significant positive correlation between *PUF60* and *AURKA* expression in bladder cancer tissues, and *PUF60* and *AURKA* expression contributed to tumor progression and malignant phenotypes in the patients with bladder cancer. Collectively, these results indicate that the *PUF60/AURKA* axis plays a key role in regulating tumorigenesis and progression of bladder cancer, and may be a potential prognostic biomarker and therapeutic target for bladder cancer patients.

## Introduction

Bladder cancer is the sixth most common cancer in men and the tenth in both sexes worldwide. There were 549,393 new cases of bladder cancer and 199,922 related deaths in 2018 worldwide (1). Even though diagnosis and treatment of bladder urothelial carcinoma have been improved in the last several decades, it remains an important public health issue worldwide due to poor management of patients (2). The tumor, node and metastasis (TNM) classification system was the most commonly used approach in risk stratification and management of bladder cancer patients. Although it has been updated many times over the last few decades, it still has limitations in predicting therapy response and outcome among bladder cancer patients (3, 4). Therefore, it is urgent to find new biomarkers for predicting the outcomes of bladder cancer, which may lead to a better management of bladder cancer patients. Recently, molecular subtypes based on the gene expression profile in bladder cancer has aroused attention worldwide, and it is promising to identify gene signatures which can better predict survival time and therapy response (5–7).

RNA splicing related proteins are a group of proteins containing RNA binding domains, which are closely involved in pre-mRNA maturation by specifically removing introns (8, 9). There are three protein families which are well-established RNA splicing factors, including serine and arginine-rich (*SR*) proteins (10), RNA binding motif (*RBM*) proteins (11) and heterogeneous nuclear ribonucleoproteins (*HNRNP*) proteins (12). Some other proteins, such as *SF3B1* (13), *PRMT5* (14), *PUF60* (15), *U2AF1* (16), and *ZRSR2* (17) also participate in the RNA splicing process. It has been reported that abnormal expression or mutations of RNA splicing proteins are widely observed in human diseases, especially in cancers (18–20). However, the exact roles of RNA splicing proteins in bladder cancer development and progression haven’t been systematically elucidated yet. It remains to be further investigated whether there are specific RNA spicing proteins playing an essential role in bladder cancer development.

Poly(U) binding splicing factor 60 (*PUF60*), also known as FUSE-binding protein-interacting repressor (*FIR*), is a nucleic acid-binding protein that plays a role in a variety of nuclear processes, including pre-mRNA splicing and transcriptional regulation (21, 22). Overexpression of *PUF60* has been reported to be closely associated with the development and progression of multiple cancers, including colon cancer (23–25), hepatocellular carcinoma (26), non-small cell lung cancer (27), breast cancer (28), and esophageal cancer (29, 30). Abnormal expression, mutation or truncation of *PUF60* were widely reported in congenital diseases associated with intellectual disability, heart defects and short stature (31–34). In summary, *PUF60* plays an important role in human diseases, including cancer and congenital diseases. However, it remains unveiled its role in the initiation, progression and prognosis of bladder cancer.

Aurora kinase A (*AURKA*) is a member of aurora kinase family, which perform essential functions during cell division (35). *AURKA* is mainly associated with the spindle poles during mitosis, where it is required for centrosome separation and maturation (36). In the past several decades, emerging studies have proved that *AURKA* plays an important role in tumor development and progression (37). Specific inhibitors targeting *AURKA* has been developed and shown promising prospect recently due to the prominent role of *AURKA* in tumor progression (38–40). In bladder cancer, inhibiting *AURKA* by its specific inhibitors could decrease cell proliferation (41), induce apoptosis (42, 43) and cause cell cycle arrest (44).

Our TCGA data analysis identified *PUF60* as one of the most differentially expressed genes among the 97 well-established RNA splicing factor genes. In this study, we investigated the potential association between *PUF60* expression and clinicopathological characteristics in bladder cancer and analyzed the potential of *PUF60* to be a new biomarker for malignant phenotypes and poor prognosis in bladder cancer. In addition, we identified *AURKA* as a new downstream target of *PUF60* in bladder cancer cells, and also proved that *PUF60* promoted bladder cancer cell growth by transcriptionally upregulating *AURKA* expression. Collectively, our findings offer new insights into the understanding of the pro-tumorigenic role of *PUF60* and its underlying mechanism involved in bladder cancer growth, and suggest that the *PUF60/AURKA* axis may provide prognostic biomarkers and therapeutic targets for bladder cancer patients.

## Materials and Methods

### Cell Culture, Antibodies and Chemicals

The human bladder cancer cell lines (5637, UM-UC-3, T24, Biu87, J82) were obtained from American Type Culture Collection (ATCC, Manassas, VA, United States) and cultured in RPMI-1640 (Invitrogen, Carlsbad, CA, United States) supplemented with 10% fetal bovine serum,100 unit/ml penicillin, and 100 μg/ml streptomycin. Cells were maintained in an incubator with a humidified atmosphere of 95% air and 5% CO_2_ at 37°C.

Anti-*PUF60* antibody was purchased from Invitrogen (Carlsbad, CA, United States), anti-*AURKA* antibody, anti-*GAPDH* and secondary rabbit antibody from Proteintech (Wuhan, China). *AURKA* inhibitor was purchased from Selleck (Shanghai, China).

### Streptavidin-Agarose Pulldown Assay

The binding of *PUF60* to *AURKA* promoter was confirmed by streptavidin-agarose pulldown assay as previously described. Briefly, 800 ng nuclear proteins from human bladder cancer cell lines were incubated with 8 μg biotin-labeled double-stranded DNA probes of *AURKA* promoter from −1239 to +43 and 8 μl streptavidin-agarose beads (Sigma-Aldrich) at 4°C overnight. The mixture was then centrifuged at 500 × *g* to pull down the DNA-protein complex. Precipitated proteins were separated by SDS-PAGE and transferred onto polyvinylidene difluoride (PVDF) membranes. The membranes were incubated with primary antibody and horseradish peroxidase-conjugated secondary antibody, and proteins were then detected using the ECL chemiluminescence system (Pierce, Rockford, IL, United States).

### Western Blotting

Briefly, cells were collected and lysed by RIPA buffer (150 mM NaCl, 0.5% EDTA, 50 mM Tris, 0.5% NP40) and centrifuged for 15 min at 12,000 rpm and 4°C. 50 μg of harvested total proteins were separated by SDS-PAGE and transferred onto polyvinylidene difluoride (PVDF) membranes. The membranes were incubated with primary antibody and horseradish peroxidase-conjugated secondary antibody, and proteins were then detected using the ECL chemiluminescence system (Pierce, Rockford, IL, United States).

### Luciferase Reporter Assay

Briefly, 5637 cells were plated in 24-well plates at a density of 1.0 × 10^5^ cells per well then transfected with 483 ng of promoter-luciferase plasmid and 17 ng of pRL-CMV. The luciferase activity was measured using a Dual-Luciferase Assay kit (Promega) 48 h after transfection. Four replicative wells were measured. The primers used for cloning the indicated promoter regions are listed in Supplementary Table 1.

### Immunohistochemistry (IHC)

Bladder cancer tissue microarrays with 56 samples were purchased from Outdo Biotech Co., Ltd. (Shanghai, China). Bladder cancer tissue microarrays with 13 pairs of samples were purchased from Alenabio Biotechnology Co., Ltd. (Xi’an, China). The primary antibodies against *PUF60* were diluted 1:100, and then incubated at 4°C overnight in a humidified container. After three washes with PBS, the tissue slides were treated with a non-biotin horseradish peroxidase detection system according to manufacturer’s instructions (Dako). IHC scores were evaluated. IHC H-score = Σ(PI × I) = (percentage of cells of weak intensity × 1) + (percentage of cells of moderate intensity × 2) + (percentage of cells of strong intensity × 3).

### RNA Extraction and qRT-PCR

Briefly, total RNA was extracted using RaPure Total RNA Micro Kit (Magen, Guangzhou, China). First-strand cDNA was synthesized using HiScript II One Step RT-PCR Kit (Vazyme, Nanjing, China). The primers used to amplify the indicated genes are listed in Supplementary Table 1. Real time q-PCR was performed using ChamQ SYBR qPCR Master Mix (Vazyme, Nanjing, China) following instructions.

### Cell Viability Assay

Cells were seeded in 96-well plates (10,000 cells/well) 24 h after *PUF60* siRNA transfection. Cell viability was assessed by the MTS assay (Promega, Madison, WI, United States) 72 h after transfection. Cell viability of stable cell lines with *PUF60* overexpression was detected 48 h after plating in 96-well plates (5,000 cells/well).

### Colony Formation Assay

Bladder cancer cell lines 5637 or T24 were seeded at a density of 800 cells per well in 6-well plates 24 h after *PUF60* siRNA transfection and cultured for 10–14 days. The colonies were then stained with 1% crystal violet and counted. All experiments were performed with three independent trials.

### siRNA and Plasmid Construction

The sequences targeting *PUF60*, 5′-UCAAGAGUGUGCUGGU GAA-3′, 5′-GCUACGGCUUCAUUGAGUA-3′ and negative control siRNA were synthesized by GenePharma Co., Ltd. (Suzhou, China). Transfection was performed according to the manufacturer’s instructions using Lipofectamine RNAiMAX transfection reagent (Invitrogen) and 50 nM siRNA.

For overexpression of *PUF60* in bladder cancer cell lines, *PUF60 or AURKA* was cloned into the pSIN-EF2-puro vector. The PLKO.1-puro vector was used to clone the shRNAs targeting *PUF60*. The six segments of *AURKA* promoter region was cloned into the pGL3-basic vector, respectively.

### Cell Cycle Analysis

Cells were seeded into six-well plates at a density of 1 × 10^5^ cells per well. After 48 h, the wells were transfected with *PUF60* specific siRNA or overexpressing plasmid. At the end of the experiments, adherent cells were trypsinized, counted, washed, and resuspended. The cells were then pelleted and fixed by dropwise addition of 70% ice-cold ethanol at 4°C overnight. The fixed cells were washed with PBS and stained with 50 μg/mL propidium iodide, 50 μg/mL RNase I, and 0.2% Triton X-100 in the dark at 37°C for 30 min and then analyzed with flow cytometry.

### Differential Gene Expression Analysis, Functional and Pathway Enrichment Analyses

The cut-off value was ±1.5, false discovery rate (FDR)-adjusted *P* < 0.05 and fold change (FC) > 1.5 or FC < 1.5 were considered as significantly differentially expressed genes. Median value of *PUF60* expression was used to divide GSE13507 patients into *PUF60*_*high*_ and *PUF60*_*low*_ groups. To investigate functional annotations of the differential genes between *PUF60*_*high*_ and *PUF60*_*low*_ groups, we employed the Database for Annotation, Visualization and Integrated Discovery (DAVID^1^) to conduct the gene ontology (GO) and Kyoto Encyclopedia of Genes and Genomes (KEGG) analyses.

### Statistical Analysis

Data of Sanchez-Carbayo and Blaveri studies were obtained from Oncomine database. Gene expression and clinical information data of other datasets used in our analysis were obtained from TCGA database or GEO database.

Statistical analyses were performed using GraphPad Prism (version 8). Chi-square test and *t*-test or Mann Whitney test were applied for variance analysis, Spearman rank correlation method was for correlation analysis, and Kaplan–Meier analysis was for survival analysis. The cut-off value for Kaplan–Meier analysis was determined by X-tile software. Each of the statistical tests was two- tailed, and *P* < 0.05 was regarded as statistically significant.

## Results

### *PUF60* Was Highly Expressed in Bladder Cancer

**FIGURE 1:**
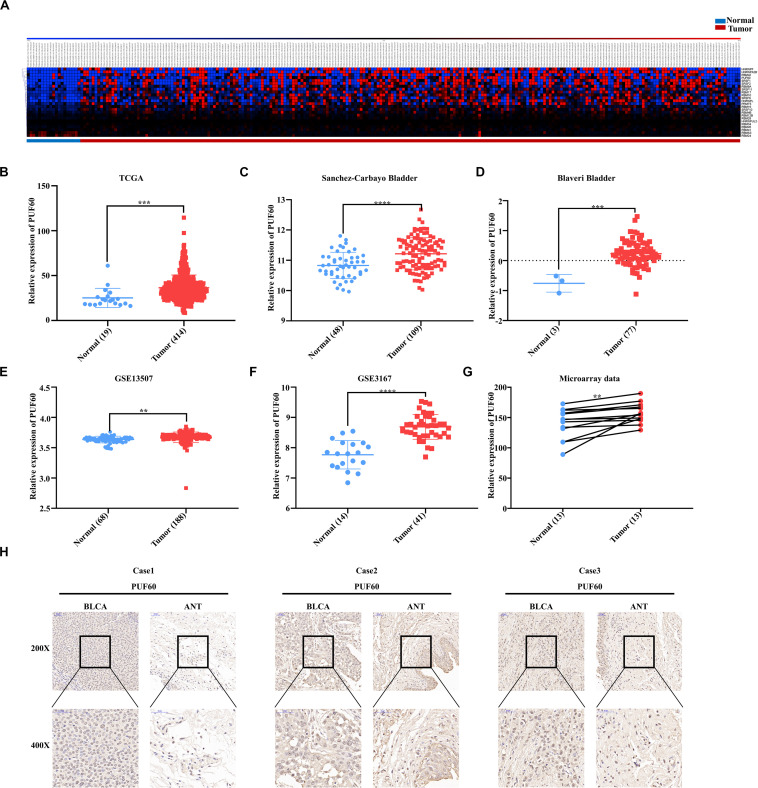
*PUF60* was highly expressed in bladder cancer. **(A)** Heat map of differentially expressed RNA splicing-related genes between normal bladder tissues and bladder cancer tissues of TCGA data. **(B)** Relative *PUF60* mRNA expression in normal and tumor tissues from TCGA (*P* = 0.0007). **(C,D)** Relative *PUF60* mRNA expression in normal and tumor tissues from two bladder cancer cohorts from Oncomine database (Sanchez-Carbayo Bladder: *P* < 0.0001; Blaveri Bladder: *P* = 0.0001). **(E,F)** Relative *PUF60* mRNA expression in normal and tumor tissues of GSE13507 and GSE3167 from GEO database (GSE13507: *P* = 0.016; GSE3167: *P* < 0.0001). **(G)** Relative expression of *PUF60* in paired normal and tumor tissues from tissue microarray data (*P* = 0.0051). **(H)** Three cases of representative IHC images of *PUF60* in bladder cancer tissues (BLCA) and adjacent normal tissues (ANT). Data was analyzed by *t*-test; **P < 0.01, ***P < 0.001, ****P < 0.0001.

We first downloaded the mRNA expression data of normal and bladder cancer tissues from TCGA database and analyzed the mRNA expression of a total of 97 RNA splicing proteins (including *SRSF* family, *RBM* family, *HNRNP* family, *SF3B1*, *PRMT5*, *PUF60*, *U2AF1*, and *ZRSR2*) between normal and carcinoma tissues. 25 genes were significantly differentially expressed (Figure 1A) (FC > 1.5, *P* < 0.05), and *HNRNPF*, *HNRNPA2B*, *RBM42*, and *PUF60* were the four most differentially expressed genes. Since the roles of *HNRNPF* and *HNRNPA2B* have been investigated in bladder cancer before, while the roles of *PUF60* in the development and progression of bladder cancer remains unclear, we set off to investigate the roles and functions of *PUF60* in bladder cancer.

Next, we confirmed the expression of *PUF60* in TCGA datasets, and found significantly higher expression of *PUF60* mRNA in carcinoma tissues compared to normal bladder tissues (Figure 1B). To further verify the expression of *PUF60* in bladder cancer, we collected the published RNA-sequencing or microarray data of bladder cancer from Oncomine database and GEO database, which include four datasets containing both normal and carcinoma tissues. We found that *PUF60* mRNA was significantly highly expressed in tumor tissues compared to normal bladder tissues in all four datasets (Figures 1C–F). To further evaluate the *PUF60* protein expression in bladder cancer, 13 bladder cancer tissues and paired adjacent normal tissues were analyzed by immunohistochemistry (IHC), confirming that bladder cancer tissues have significantly higher *PUF60* protein expression compared to paired normal tissues (Figures 1G,H). This result was consistent with our analysis of *PUF60* mRNA expression in public databases.

### High *PUF60* Expression Was Associated With Malignant Phenotypes in Bladder Cancer

**FIGURE 2:**
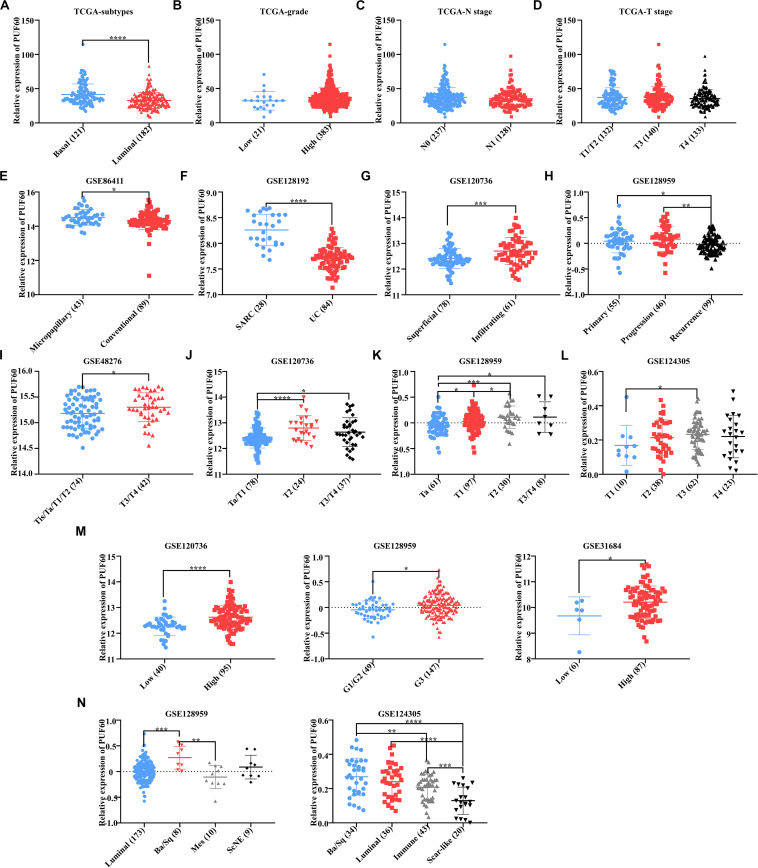
High *PUF60* expression was associated with malignant phenotypes in bladder cancer. **(A)** Relative *PUF60* mRNA expression in basal-like and luminal-like bladder cancer tissues from TCGA data (*P* < 0.0001). **(B)** Relative *PUF60* mRNA expression in low and high grade bladder cancer tissues from TCGA data (*P* = 0.1219). **(C)** Relative *PUF60* mRNA expression in lymph node negative (N0) and positive (N1) tissues from TCGA data (*P* = 0.1821). **(D)** Relative *PUF60* mRNA expression of different T stage tissues from TCGA data (*P* = 0.3815). **(E)** Relative *PUF60* mRNA expression in bladder micropapillary urothelial carcinoma and conventional urothelial carcinoma of GSE86411 data (*P* = 0.0381). **(F)** Relative *PUF60* mRNA expression in sarcomatoid urothelial bladder cancer (SARC) and conventional bladder urothelial carcinoma (UC) of GSE128192 data (*P* < 0.0001). **(G)** Relative *PUF60* mRNA expression in superficial and infiltrating bladder cancer of GSE120736 (*P* = 0.0002). **(H)** Relative *PUF60* mRNA expression in primary, progressive and recurrent bladder cancer of GSE128959 data (Recurrence vs. Primary: *P* = 0.0323; Recurrence vs. Progressive: *P* = 0.0019). **(I–L)** Relative *PUF60* mRNA expression in different T stage tissues of four independent GEO datasets: GSE48276 **(I)**, GSE120736 **(J)**, GSE128959 **(K)**, GSE124305 **(L)** (GSE48276: *P* = 0.0359; GSE120736: T2 vs. Ta/T1: *P* < 0.0001, T3/T4 vs. Ta/T1: *P* = 0.0115; GSE128959: T1 vs. Ta: *P* = 0.0246, T2 vs. Ta: *P* = 0.0007, T3/T4 vs. Ta: *P* = 0.0416, T2 vs. T1: *P* = 0.0472; GSE124305: *P* = 0.0425). **(M)** Relative *PUF60* mRNA expression in low and high grade bladder cancer from three independent GEO datasets (**left to right:** GSE120736, GSE128959, and GSE31684) (GSE120736: *P* < 0.0001; GSE128959: *P* = 0.0108; GSE31684: *P* = 0.0490). **(N)** Relative *PUF60* mRNA expression in different molecular subtypes of GSE128959 **(left)** and GSE124305 **(right)**; luminal, Luminal-like subtype bladder cancer; Ba/Sq, Basal/Squamous-like subtype bladder cancer; Mes, Mesenchymal-like subtype bladder cancer; ScNE, Small-cell/Neuroendocrine-like subtype bladder cancer; Immune, Immune subtype bladder cancer; Scar-like, Scar-like subtype bladder cancer (GSE128959: luminal vs. Ba/Sq: *P* = 0.0004, Ba/Sq vs. Mes: *P* = 0.0027; GSE124305: Ba/Sq vs. Scar-like: *P* < 0.0001, Ba/Sq vs. Immune: *P* = 0.0049, Luminal vs. Scar-like: *P* < 0.0001, Immune vs. Scar-like: *P* = 0.0003). Data was analyzed by *t*-test; *P < 0.05, **P < 0.01, ***P < 0.001, ****P < 0.0001.

Our previous work proved that *PUF60* was highly expressed in bladder cancer, but it was unclear the association between *PUF60* mRNA expression and bladder cancer phenotypes, such as histopathological type, T stage, grade and molecular subtypes. Hence, we analyzed the association between *PUF60* mRNA expression and molecular subtypes, grade, N stage or T stage of TCGA bladder cancer expression data. We found that the mRNA expression of *PUF60* was significantly higher in basal bladder cancer tissues, which was a malignant molecular subtype with poor survival (Figure 2A). In contrast, we didn’t find any association between *PUF60* mRNA expression and other pathological phenotypes (Figures 2B–D). To further investigate the mRNA expression profile of *PUF60* in different phenotypes of bladder cancer, we searched the GEO database for bladder cancer datasets that have over 90 samples as well as clinical and histopathological information of patients. Seven datasets from different studies (GSE86411, GSE128192, GSE120736, GSE128959, GSE48276, GSE124305, and GSE31684) were selected. Our analysis of GSE86411 and GSE128192 revealed that *PUF60* mRNA was significantly highly expressed in micropapillary bladder cancer and Sarcomatoid urothelial bladder cancer (SARC), which displayed a high propensity for distant metastasis and were associated with short survival (Figures 2E,F), compared to conventional urothelial carcinoma. We also analyzed the *PUF60* mRNA expression in superficial and infiltrating bladder urothelial carcinoma of GSE120736 dataset, finding that infiltrating tissues tended to have higher *PUF60* mRNA expression (Figure 2G). Intriguingly, when we investigated the *PUF60* mRNA expression in primary, progressive and recurrent bladder cancer tissues of GSE128959 dataset, we found that primary or progressive bladder cancer had higher *PUF60* mRNA expression than recurrent cancer (Figure 2H). This suggests that *PUF60* may play an important role in bladder cancer initiation and progression. Moreover, we found *PUF60* mRNA was highly expressed in advanced T stage bladder cancer in most of datasets (Figures 2I–L) and high grade bladder cancer also tended to have higher *PUF60* mRNA expression (Figure 2M). Unfortunately, there weren’t datasets with enough patient information regarding N stage and M stage. Finally, our analysis of *PUF60* mRNA expression in different molecular subtypes of bladder cancer showed that basal types had higher *PUF60* mRNA expression (Figure 2N), which was consistent with our previous analysis in TCGA dataset. These results indicated that *PUF60* are closely associated with most of malignant phenotypes in bladder cancer, and it was a potential molecular marker for malignant behavior in bladder cancer.

### *PUF60* Predicted Unfavorable Outcomes in Bladder Cancer Patients

**FIGURE 3:**
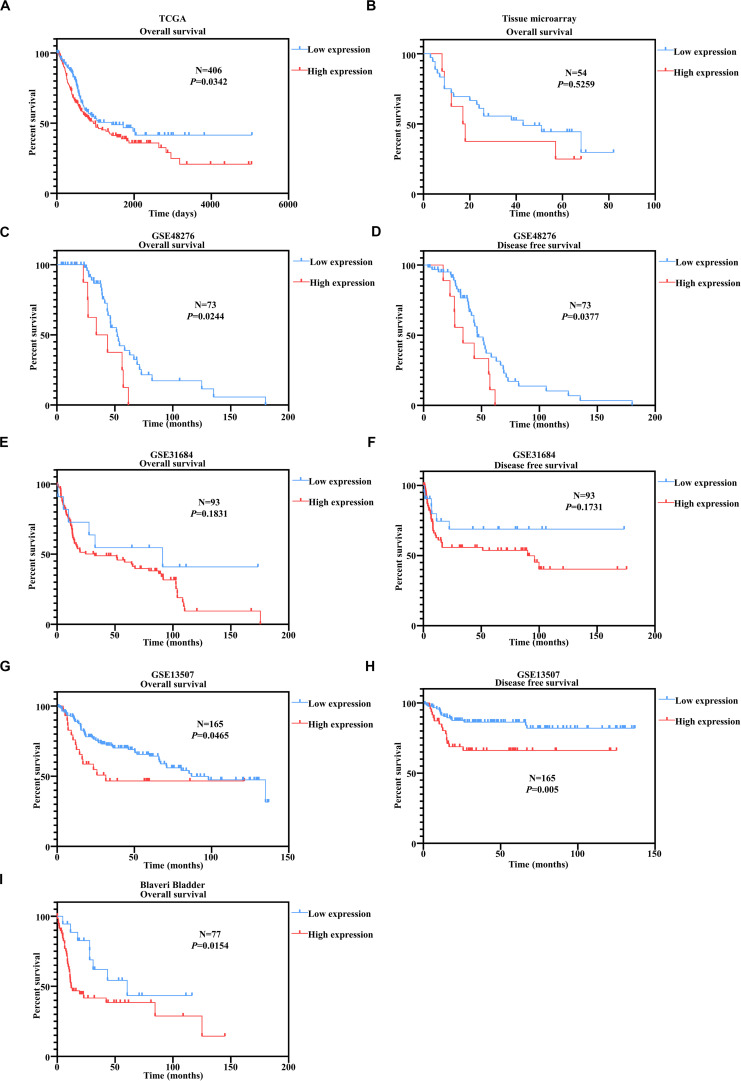
*PUF60* predicted unfavorable outcomes in bladder cancer patients. **(A)** Kaplan–Meier analysis of overall survival according to the *PUF60* mRNA expression from TCGA data. **(B)** Kaplan–Meier analysis of overall survival according to the *PUF60* protein expression from tissue microarray data. **(C,D)** Kaplan–Meier analysis of overall **(left)** and disease free survival **(right)** according to the *PUF60* mRNA expression from GSE48276 data. **(E,F)** Kaplan–Meier analysis of overall **(left)** and disease free survival **(right)** according to the *PUF60* mRNA expression from GSE31684 data. **(G,H)** Kaplan–Meier analysis of overall **(left)** and disease free survival **(right)** according to the *PUF60* mRNA expression from GSE13507 data. **(I)** Kaplan–Meier analysis of overall **(left)** and disease free survival **(right)** according to the *PUF60* mRNA expression from Blaveri bladder studies.

We have proved above that *PUF60* mRNA was highly expressed and associated with malignant phenotypes in bladders. To examine whether *PUF60* could serve as a prognostic biomarker for bladder cancer patients. We conducted Kaplan–Meier survival analysis according to the *PUF60* mRNA expression of TCGA dataset in bladder cancer patients. We found that patients with high mRNA expression of *PUF60* had significant shorter overall survival time (Figure 3A). To confirm the prognostic value of *PUF60* protein expression for bladder cancer patients, we next conducted Kaplan–Meier survival analysis of our tissue microarray data according to the *PUF60* protein expression score. The result showed that patients with higher *PUF60* protein expression had a poorer overall survival (Figure 3B), consistent with our analysis of TCGA data. The difference between low and high *PUF60* protein expression is not significant though, probably due to our limited number of samples. We also analyzed the association between *PUF60* mRNA expression and clinical pathological characteristics of patients, but no significant difference was found (Table 1). To further prove our conclusion, we conducted the Kaplan–Meier survival analysis in several GEO datasets with the survival information. Patients with high *PUF60* mRNA expression are prone to have shorter overall survival time in three out of the four datasets (Figures 3C, E, G, I). In addition, we analyzed the disease free survival according to the *PUF60* mRNA expression in three independent GEO datasets. The result revealed that patients with high *PUF60* mRNA expression had significantly shorter survival time in two out of the three cohorts (Figures 3D,F,H), which was consistent with the analysis of overall survival. These results supported that *PUF60* was a potential prognostic biomarker for bladder cancer patients.

**TABLE 1:** Correlation between PUF60 and clinical pathology characteristics in bladder cancer of tissue microarray data.

Variable	NO.	PUF60		*X*^2^	*P* Valve
		Low expression	High expression		
**Age**					
<60	12	3 (25.0%)	9 (75.0%)	2.363	0.124
>60	42	21 (50.0%)	21 (50.0%)		
**Gender**					
Female	8	2 (25.0%)	6 (75.0%)	1.438	0.23
Male	46	22 (47.8%)	24 (52.2%)		
**T stage**					
Tis	5	3 (60.0%)	2 (40.0%)	0.997	0.91
1	10	5 (50.0%)	5 (50.0%)		
2	14	6 (42.9%)	8 (57.1%)		
3	21	8 (38.1%)	13 (61.9%)		
4	2	1 (50.0%)	1 (50.0%)		
**N stage**					
0	33	15 (45.5%)	18 (54.5%)	0.042	0.837
1	6	3 (50.0%)	3 (50.0%)		
**AJCC stage**					
Ois	3	2 (66.7%)	1 (33.3%)	7.061	0.133
I	6	3 (50.0%)	3 (50.0%)		
II	12	2 (16.7%)	10 (83.3%)		
III	12	8 (66.7%)	4 (33.3%)		
IV	7	4 (57.1%)	3 (42.9%)		
**Pathological grade**					
II	16	5 (31.3%)	11 (68.8%)	1.603	0.205
II–III/III	38	19 (50.0%)	19 (50.0%)		

### *PUF60* Promotes Bladder Cancer Cell Growth and Cell Cycle Progression

**FIGURE 4:**
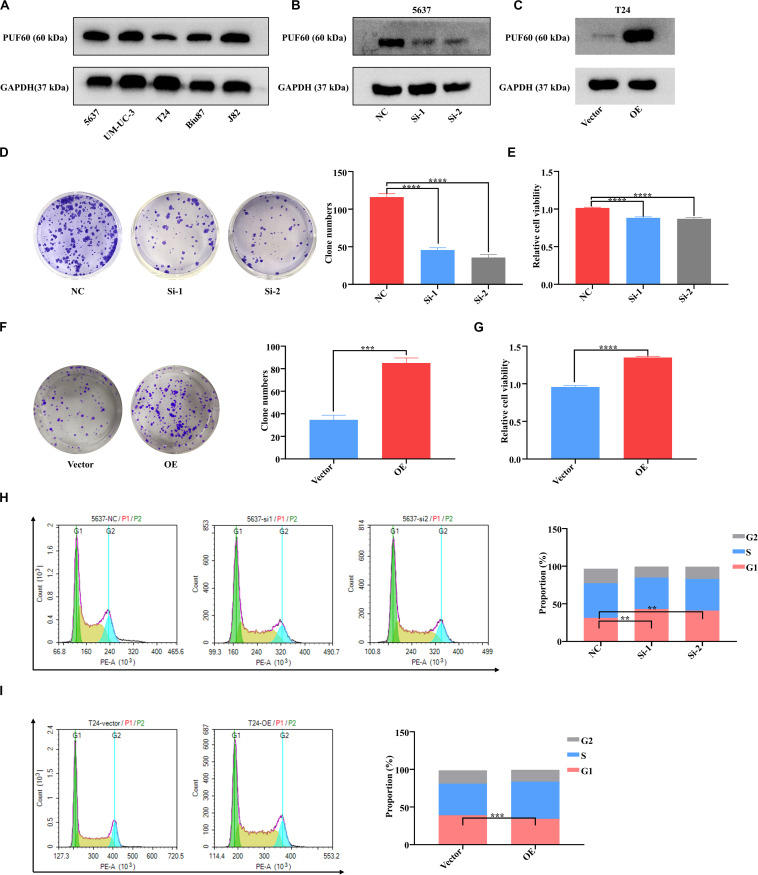
*PUF60* promoted bladder cancer cell growth and cell cycle progression. **(A)** Endogenous expression of *PUF60* was detected by western blot in different bladder cancer cell lines. **(B)** *PUF60* expression was knocked down by its specific siRNAs in 5637 cells and detected by western blot. **(C)** *PUF60* was overexpressed in T24 cells and detected by western blot. **(D,E)** Knockdown of *PUF60* inhibited the clonogenicity **(D)** and cell viability **(E)** of 5637 cells (**D:** NC vs. Si-1: *P* < 0.0001, NC vs. Si-2: *P* < 0.0001; **E:** NC vs. Si-1: *P* < 0.0001, NC vs. Si-2: *P* < 0.0001). **(F,G)** Overexpression of *PUF60* promoted the clonogenicity **(F)** and cell viability **(G)** of T24 cells (**F:** OE vs. vector: *P* = 0.0001; **G:** OE vs. vector: *P* < 0.0001). **(H)** Knockdown of *PUF60* inhibited cell cycle progression in 5637 cells (NC vs. Si-1: *P* = 0.0018, NC vs. Si-2: *P* < 0.0027). **(I)** Overexpression of *PUF60* promoted cell cycle progression in T24 cells (OE vs. vector: *P* = 0.0005). Data was analyzed by *t*-test; **P < 0.01, ***P < 0.001, ****P < 0.0001.

To investigate the biological functions of *PUF60* in bladder cancer, we detected the protein expression of *PUF60* in five bladder cancer cell lines (5637, UM-UC-3, T24, Biu87, J82) by western blot (Figure 4A). We then chose 5637 cells with high *PUF60* expression to knock down its expression (Figure 4B), and T24 cells with low *PUF60* expression to overexpress its expression (Figure 4C). We found that knockdown of *PUF60* significantly inhibited the proliferation and clonogenicity of 5637 cells (Figures 4D,E), whereas overexpression of *PUF60* significantly increased the proliferation and clonogenicity in T24 cells (Figures 4F,G). Furthermore, we found that knockdown of *PUF60* caused G1/S arrest in 5637 cells (Figure 4H), while overexpression of *PUF60* promoted cell cycle progression in T24 cells (Figure 4I).

### Associations Between Genome-Wide Expression Profiles and *PUF60* Expression

**FIGURE 5:**
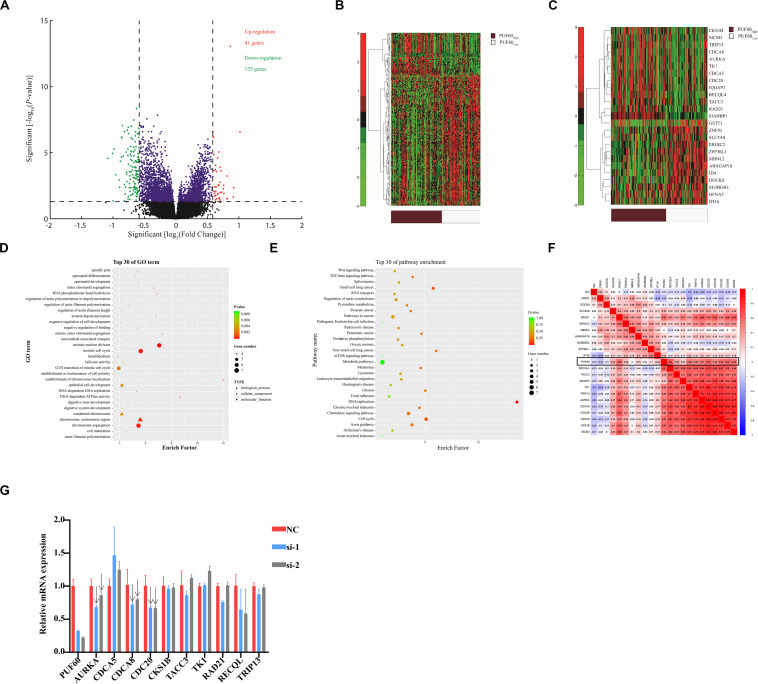
Associations between genome-wide expression profiles and *PUF60* expression. **(A)** Volcano plot of differentially expressed gene profiles between *PUF60*_*high*_ and *PUF60*_*low*_. **(B)** Expression heat map of *PUF60*-associated genes between *PUF60*_*high*_ and *PUF60*_*low*_. **(C)** Expression heat map of differentially expressed genes closely related to bladder cancer development. **(D,E)** GO and KEGG analyses of differentially expressed genes between *PUF60*_*high*_ and *PUF60*_*low*_. **(F)** Correlation between *PUF60* and genes identified relevant to bladder cancer development from TCGA data. **(G)** mRNA levels of *PUF60* and its possible downstream genes were detected by real time q-PCR in cells with *PUF60* knockdown.

To further clarify the potential underlying mechanism of *PUF60*-regulated bladder cancer cell growth, we selected GSE1357 dataset, which included the most samples of primary bladder cancer and intact clinical information of patients in all the datasets, to conduct gene expression profile analysis. The median value of *PUF60* mRNA expression was used to divide patients into *PUF60*_*high*_ and *PUF60*_*low*_ groups, and a total of 40 up-regulated and 125 down-regulated genes were identified to be significantly associated with *PUF60* mRNA expression (FDR-adjusted *P* < 0.05 and FC > 1.5 or FC < 1.5, Figure 5A). Aberrantly expressed genes were displayed/identified in an expression heat map (Figure 5B). Among the up-regulated genes in the *PUF60*_*high*_ group, 12 out 40 were those promoting the proliferation and progression of bladder cancer or serving as independent unfavorable biomarkers for patients, including *MCM2* (45), *CKS1B* (46), *CDCA8* (47), *TK1* (48), *RAD21* (49), *AURKA* (42, 44), *CDCA5* (50), *TRIP13* (51), *TACC3* (52), *IQGAP3* (53–55), *CDC20* (56, 57), *RECQL4* (58). On the other hand, some of the down-regulated genes included the well-established tumor-suppressing genes in cancers, such as *IFI16* (59, 60), *ERGIC2* (61–63), *SLC5A8* (64–72) (Figure 5C). These results indicated that *PUF60* may play an important role in the initiation and progression of bladder cancer through up-regulating some oncogenes while down-regulating certain tumor-suppressing genes.

To elucidate the potential molecular pathways that involve *PUF60* in the development and progression of bladder cancer, we conducted GO terms and KEGG enrichments analyses based on differential genes in the two groups. The results show that biological processes relevant to cell division, including meiotic nuclear division, meiotic cell cycle and chromosome segregation, were among the top 10 in GO terms analysis (Figure 5D). KEGG analysis also showed that DNA replication and cell cycle were among the top differential pathways (Figure 5E). These results were consistent with our cellular experiments *in vitro*. All these evidences indicated that *PUF60* might be one of the dominant factors that mediate the initiation and progression of various cancers by influencing some key biological processes or pathways. To further validate the correlation between *PUF60* and the differentially expressed genes identified above in the GSE13507 dataset, we analyzed the correlation between *PUF60* and genes identified above using TCGA expression data, and found that 22 out of 25 genes were also significantly correlated with *PUF60* (Figure 5F). To test whether PUF60 can regulate the expression of genes identified above, we knocked down *PUF60* expression by its specific siRNAs in bladder cancer cell line 5637, and then detected the mRNA levels of 10 genes that were significantly associated with *PUF60* in both TCGA and GES13507 datasets. We found mRNA expression of *AURKA*, *CDCA8*, *CDC20* were decreased upon knockdown of *PUF60* by its specific siRNA, indicating that they are the potential downstream targets of *PUF60* in bladder cancer (Figure 5G, indicated by the arrows).

### *PUF60* Promoted Bladder Cancer Cell Growth via Transcriptionally Upregulating *AURKA* Expression

**FIGURE 6:**
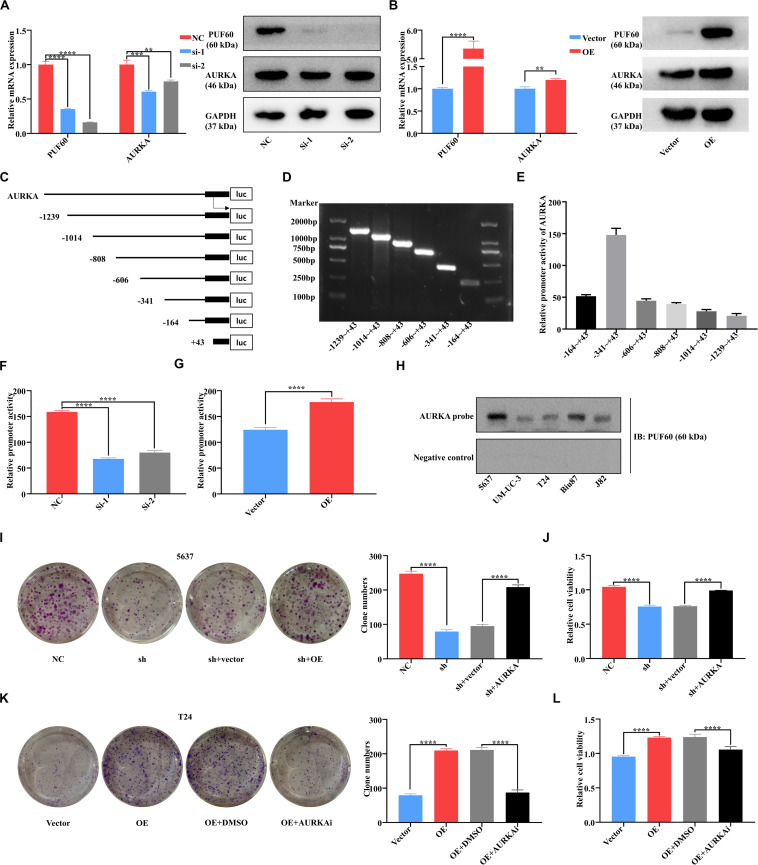
*PUF60* promoted bladder cancer cell growth via transcriptionally upregulating *AURKA* expression. **(A)** *PUF60* and *AURKA* expression was detected by RT-qPCR and western blot in 5637 cells with *PUF60* knocked down (*PUF60*: NC vs. Si-1: *P* < 0.0001, NC vs. Si-2: *P* < 0.0001; *AURKA*: NC vs. Si-1: *P* = 0.0005, NC vs. Si-2: *P* < 0.0032). **(B)** *PUF60* and *AURKA* expression was detected by RT-qPCR and western blot in T24 cells overexpressing *PUF60* (*PUF60*: OE vs. vector: *P* < 0.0001; *AURKA*: OE vs. vector: *P* = 0.0033). **(C)** Ideograph of different segments of *AURKA* promoter. **(D)** Different segments of *AURKA* promoter were amplified by PCR using specific primers, and PCR products were detected by agarose gel electrophoresis. **(E)** Relative promoter activity of different segments of *AURKA* promoter measured by dual luciferase assay. **(F)** Relative activity of *AURKA* promoter was measured after knockdown of *PUF60* in 5637 cells (NC vs. Si-1: *P* < 0.0001, NC vs. Si-2: *P* < 0.0001). **(G)** Relative activity of *AURKA* promoter was measured after overexpression of *PUF60* in T24 cells (OE vs. vector: *P* < 0.0001). **(H)** Binding of *PUF60* on the 5′-biotin labeled *AURKA* promoter probe or a control non-specific probe was detected by Western blot using anti-*PUF60* antibody. **(I,J)** Knockdown of *PUF60* inhibited the clonogenicity **(I)** and viability **(J)** of 5637 cells, which was reversed by *AURKA* overexpression (**I:** NC vs. sh: *P* < 0.0001, sh + vector vs. sh + *AURKA*: *P* < 0.0001; **J:** NC vs. sh: *P* < 0.0001, sh + vector vs. sh + *AURKA*: *P* < 0.0001). **(K,L)** Overexpression of *PUF60* promoted the clonogenicity **(K)** and viability **(L)** of T24 cells, which was reversed by *AURKA* inhibitor (**K:** OE vs. vector: *P* < 0.0001, OE + DMSO vs. OE + *AURKA*i: *P* < 0.0001; **L:** OE vs. vector: *P* < 0.0001, OE + DMSO vs. OE + *AURKA*i: *P* < 0.0001). Clonogenicity was determined by colony formation assay. Viability was measured by MTS assay. Data was analyzed by *t*-test; **P < 0.01, ***P < 0.001, ****P < 0.0001.

Considering the well-acknowledged vital roles of *AURKA* in bladder cancer development and progression, we conjectured that *AURKA* was the most potential target of *PUF60*. To confirm our hypothesis, we knocked down *PUF60* expression by its specific siRNAs in 5637 cells, which significantly decreased the mRNA and protein expression of *AURKA* (Figure 6A). In contrast, overexpression of *PUF60* in T24 cells significantly increased the expression of *AURKA* (Figure 6B). Next, we analyzed the association between mRNA expression of *PUF60* or *AURKA* and the clinical pathological characteristics in GSE13507 datasets (Table 2), showing that higher *AURKA* mRNA expression was significantly associated with age, gender, AJCC stage and grade of patients.

**TABLE 2:** Correlation between PUF60/AURKA and clinical pathology characteristics in bladder cancer.

Variable	NO.	PUF60		*X*^2^	*P* Valve	AURKA		*X*^2^	*P* Valve
		Low expression	High expression			Low expression	High expression		
**Age**									
<60	42	23 (54.8%)	19 (45.2%)	0.578	0.447	31 (73.8%)	11 (26.2%)	7.93	0.005
>60	123	59 (48.0%)	64 (52.0%)			60 (48.8%)	63 (51.2%)		
**Gender**									
Female	30	12 (40.0%)	18 (60.0%)	1.379	0.24	9 (30.0%)	21 (70.0%)	9.378	0.002
Male	135	70 (51.9%)	60 (48.1%)			82 (60.7%)	53 (39.3%)		
T stage									
Ta	24	15 (62.5%)	9 (37.5%)	5.88	0.208	19 (79.2%)	5 (20.8%)	23.631	<0.0001
1	80	40 (50.0%)	40 (50.0%)			50 (62.5%)	30 (37.5%)		
2	31	16 (51.6%)	15 (48.4%)			15 (48.4%)	16 (51.6%)		
3	19	5 (26.3%)	14 (73.7%)			2 (10.5%)	17 (89.5%)		
4	11	6 (54.5%)	5 (45.5%)			5 (45.5%)	6 (54.5%)		
**N stage**									
N−	149	75 (50.3%)	74 (49.7%)	0.286	0.593	86 (57.7%)	63 (42.3%)	2.513	0.113
N+	14	6 (42.9%)	8 (57.1%)			5 (35.7%)	9 (64.3%)		
M stage									
M0	158	79 (50.0%)	79 (50.0%)	<0.0001	1	88 (55.7%)	70 (44.3%)	0.076	0.783
M1	6	3 (50.0%)	3 (50.0%)			3 (50.0%)	3 (50.0%)		
**AJCC stage**									
0a	23	15 (65.2%)	8 (34.8%)	4.422	0.352	19 (82.6%)	4 (17.4%)	20.577	<0.001
I	80	40 (50.0%)	40 (50.0%)			50 (62.5%)	30 (37.5%)		
II	26	13 (50.0%)	13 (50.0%)			12 (46.2%)	14 (53.8%)		
III	19	7 (36.8%)	12 (63.2%)			4 (21.1%)	15 (78.9%)		
IV	16	6 (37.5%)	10 (62.5%)			6 (37.5%)	10 (62.5%)		
**Pathological grade**									
low	105	60 (57.1%)	45 (42.9%)	6.404	0.011	79 (75.2%)	26 (24.8%)	47.101	<0.0001
high	60	22 (36.7%)	38 (63.3%)			12 (20.0%)	48 (80.0%)		

Recent studies reported that RNA binding proteins were widely associated with gene transcriptional regulation, leading us to examine whether *PUF60* upregulated *AURKA* expression by transcriptional activation. We constructed the luciferase reporter plasmids that have six different segments of the *AURKA* promoter (Figures 6C,D). The luciferase reporter assay showed that −341 ∼+43 was the core promoter of *AURKA* (Figure 6E), so we chose this plasmid for following validation assays. We then knocked down *PUF60* expression in 5637 cells, and found that it significantly decreased the *AURKA* promoter activity (Figure 6F), while overexpression of *PUF60* in T24 cells significantly increased the *AURKA* promoter activity (Figure 6G). To further validate that *PUF60* transcriptionally activated *AURKA* expression, we conducted streptavidin-agarose pulldown assay, which showed that there was obvious binding of *PUF60* at the *AURKA* promoter (Figure 6H). The results proved that *PUF60* regulated *AURKA* expression by specifically binding to its promoter, thus activating its transcription.

To corroborate that *PUF60* indeed regulated bladder cancer cell growth by mediating *AURKA* expression, we conducted the expression rescue experiments. We found that knockdown of *PUF60* significantly inhibited 5637 cells growth, while overexpression of *AURKA* could partially reverse such an effect (Figures 6I,J). Consistently, overexpression of *PUF60* significantly increased T24 cells growth, which was partially reversed by *AURKA* specific inhibitor (Figures 6K,L). These results suggested that *PUF60* regulated bladder cancer cell growth by transcriptionally activating *AURKA* expression.

### *AURKA* Was Highly Expressed and Positively Correlated With *PUF60* Expression in Bladder Cancer

**FIGURE 7:**
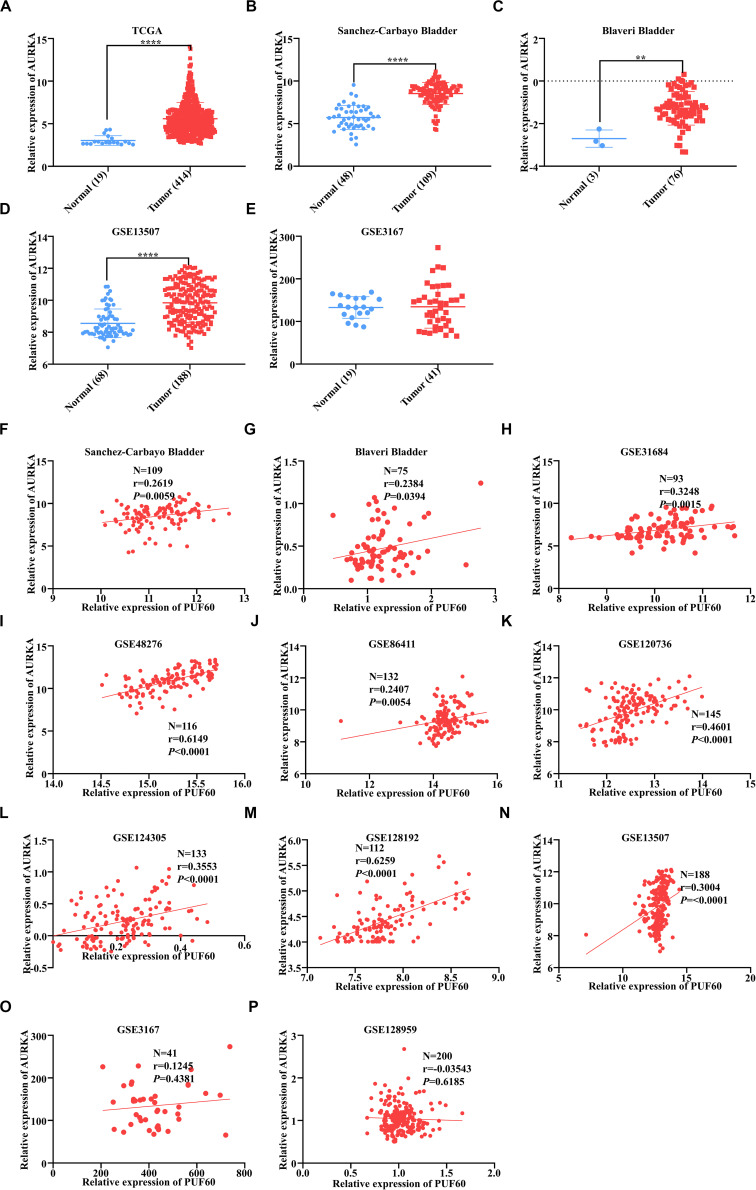
*AURKA* was highly expressed and positively correlated with *PUF60* expression in bladder cancer. **(A–E)** Relative *AURKA* mRNA expression between normal and tumor tissues in TCGA data **(A)**, Sanchez-Carbayo bladder study **(B)**, Blaveri bladder study **(C)**, GEO13507 **(D)**, and GEO3167 data **(E)** (**A:** *P* < 0.0001; **B:** *P* < 0.0001; **C:** *P* = 0.0026; **D:** *P* < 0.0001; **E:** *P* = 0.8837). **(F–P)** The correlation between *AURKA* and *PUF60* mRNA expression in all datasets used before. Data was analyzed by *t*-test; **P < 0.01, ****P < 0.0001.

We analyzed the *AURKA* mRNA expression between normal and tumor tissues in our TCGA and GEO data. Four out of five datasets showed that *AURKA* mRNA was significantly highly expressed in tumor tissues (Figures 7A–E). Next, we analyzed the mRNA expression correlation between *PUF60* and *AURKA* in all datasets we used, and *AURKA* mRNA expression had significant positive correlation with *PUF60* mRNA expression in 9 out of 11 datasets (Figures 7F–P). These results are in line with the regulation relationship between *PUF60* and *AURKA*.

## Discussion

Over the past three decades, scientists have acquired a better understanding of human cancer initiation and progression with the rapid development of genome sequencing technique (73). Molecular subtypes of cancers rather than conventional clinical pathological subtypes have gained increasing attention in prognosis and treatment response prediction for patients (74). Molecular subtypes of bladder cancer have come into our sight because of its promising roles in predicting prognosis and guiding clinical treatment (5–7). It is of importance to identify genes associated with different molecular subtypes in bladder cancer.

RNA splicing proteins are a group of proteins not only involved in pre-mRNA splicing but also RNA export and transcriptional regulation. It has been reported that these proteins were widely associated with human diseases (8, 9, 18–20, 75). In the present study, we identified *PUF60* as one of the most differentially expressed genes between normal and tumor bladder tissues among the 97 RNA splicing proteins. Next, we confirmed that the protein and mRNA expression of *PUF60* were overexpressed in bladder cancer by analyzing our tissue microarray data and expression data from Oncomine database and GEO database. Furthermore, we found high *PUF60* mRNA expression was associated with more malignant histological subtypes, higher pathological grade, advanced T stage and malignant molecular subtypes in bladder cancer. Kaplan–Meier survival analysis also indicated that patients with higher *PUF60* mRNA expression are prone to have shorter survival time. To investigate its biological functions in bladder cancer cells, we conducted *in vitro* cell experiment, demonstrating that knockdown of *PUF60* inhibited bladder cancer cell growth and cell cycle progression, while overexpression had opposite effects. These results showed that *PUF60* served as an oncogene in bladder cancer. In our future study, we will perform animal experiments to verify the oncogenic role of PUF60/AURKA in bladder cancer *in vivo*. Our analysis also showed that PUF60 was associated with the malignant phenotypes of bladder cancer, and our current data indicated that PUF60 promoted cell cycle progression and growth in bladder cancer cells. It is worth investigating whether PUF60 is involved in other biological and functional processes of bladder cancer progression, such as migration, invasion and maintenance of stemness.

To clarify the underlying molecular mechanism by which *PUF60* promoted bladder cancer growth, we analyzed the association between genome-wide expression profiles and *PUF60* mRNA expression based on the data from GSE13507. *AURKA*, a vital gene involved in bladder cancer progression (41–44), was identified as a possible target of *PUF60*. A recent study indicated that pervasive chromatin-RNA binding protein interactions played an important role in the gene transcriptional regulation process beyond our expectation (76). Hence, we explored whether *PUF60* upregulated *AURKA* expression transcriptionally by binding to its promoter, which was proved by our luciferase reporter assay and streptavidin-agarose pulldown assay. We will explore the potential downstream targets of the PUF60/AURKA axis in our follow-up study, which will help explain why the knockdown of PUF60 was only partially rescued by the overexpression of AURKA. Currently, it is unclear whether the RNA splicing functions of PUF60 were involved in the regulation of AURKA expression. Moreover, though our work proved that PUF60 regulated the promoter activity of AURKA, our present data cannot distinguish whether PUF60 directly or indirectly bound to the AURKA promoter. Considering that PUF60 is not a transcription factor, we speculate that PUF60 might interact with other components of the transcription machinery to regulate AURKA expression. Noteworthily, we cannot rule out the possibility that PUF60 could influence the expression of certain transcription factors that can directly bind to the AURKA promoter to regulate its expression. Lastly, we analyzed the correlation between *PUF60* and *AURKA* expression in all datasets we used, finding significant correlation between the two genes, which further supported our conclusions.

In summary, we found that *PUF60* was highly expressed in bladder cancer cells and associated with malignant phenotypes of bladder cancer. *PUF60* promoted bladder cancer cell growth by activating *AURKA* signaling. High expression of *PUF60* and *AURKA* predicted poor prognosis in bladder cancer patients. Our findings have demonstrated that *PUF60* plays an important role in bladder cancer growth and provided new insights into the understanding of the pro-tumorigenic role of *PUF60*, indicating the *PUF60/AURKA* axis may serve as a potential clinical prognostic biomarker or a possible therapeutic target for bladder cancer.

## Data Availability Statement

All datasets presented in this study are included in the article/Supplementary Material.

## Author Contributions

QL, WD, FZ, and LH conceived the study, participated in the design of the study, and interpretation of data. QL, XA, MC, NW, SS, YL, CZ, KL, XW, TT, YP, and HQ performed the experiments. QL, FX, WD, FZ, and LH analyzed and prepared data for publication. QL, MC, WD, and LH drafted and revised the manuscript. All authors contributed to the article and approved the submitted version.

## Conflict of Interest

The authors declare that the research was conducted in the absence of any commercial or financial relationships that could be construed as a potential conflict of interest.

## Footnotes

1 https://david.abcc.ncifcrf.gov/

## Supplementary Material

TABLE S1 Primers for PCR and real-time PCR.
